# DHA does not protect *ELOVL4* transgenic mice from retinal degeneration

**Published:** 2009-06-13

**Authors:** Feng Li, Lea D. Marchette, Richard S. Brush, Michael H. Elliott, Yun-Zheng Le, Kimberly A. Henry, Ashley G. Anderson, Chao Zhao, Xufang Sun, Kang Zhang, Robert E. Anderson

**Affiliations:** 1 Department of Ophthalmology, University of Oklahoma Health Sciences Center, Oklahoma City, OK; 2 Department of Cell Biology, University of Oklahoma Health Sciences Center, Oklahoma City, OK; 3 Department of Medicine, University of Oklahoma Health Sciences Center, Oklahoma City, OK; 4 Dean A. McGee Eye Institute, Oklahoma City, OK; 5 Shiley Eye Center, University of California San Diego, San Diego, CA

## Abstract

**Purpose:**

Dominant Stargardt macular dystrophy (STGD3) is caused by several different mutations in a gene named *ELOVL4*, which shares sequence homologies with a family of genes that encode proteins involved in the ELOngation of Very Long chain fatty acids. Studies have suggested that patients with STGD3 have aberrant metabolism of docosahexaenoic acid (DHA, 22:6n3), the major polyunsaturated fatty acid (PUFA) in retinal rod outer segment membranes. We tested the effect of DHA on the progression of retinal degeneration in transgenic mice that express one of the mutations identified in STGD3.

**Methods:**

Transgenic mice expressing mutant human *ELOVL4* (TG2) were bred to mice expressing the *fat-1* protein, which can convert n6 to n3 PUFA. Mice were maintained on an n3-deficient diet containing 10% safflower oil (SFO, enriched in n6 PUFA; n6/n3=273) so that four experimental groups were produced that differed only in levels of n3 PUFA and expression of the *hELOVL4* transgene. These groups were identified by genotyping and named Fat1^+^/TG2^+^, Fat1^–^/TG2^+^, Fat1^+^/TG2^–^, and Fat1^–^/TG2^–^. All were continued on the SFO diet for 4 to 16 weeks such that those not expressing Fat1 would be deficient in n3 fatty acids. At both time points, animals were analyzed for retinal function by electroretinography (ERG), photoreceptor cell viability by outer nuclear layer (ONL) thickness measurements, fatty acid profiles in several tissues, and rhodopsin levels.

**Results:**

Mice expressing the *fat-1* transgene had significantly higher levels of n3 PUFA, primarily DHA, in retina, liver, and plasma lipids at 4 and 16 weeks of age. Retinal DHA levels in *fat-1* mice were twice those of controls. By 16 weeks of age, mice expressing the mutant *hELOVL4* transgene had a significantly greater loss of photoreceptor cells, reduced ERG amplitudes, and lower rhodopsin levels than control mice. There was no effect of retinal fatty acids on the rate of degeneration of retinas expressing the *ELOVL4* transgene.

**Conclusions:**

We found no evidence that high levels of DHA in retinal membranes protected photoreceptor cells expressing mutant *ELOVL4* from retinal degeneration. We conclude that DHA is not beneficial for the treatment of retinal degeneration in this animal model of human STGD3 macular dystrophy.

## Introduction

In 2001, a 5 bp deletion in a novel gene was identified in several families with autosomal dominant Stargardt-like macular dystrophy (STGD3). The gene was found to share homologies throughout the entire sequence with the family of fatty acid elongases (*ELO)* [1,2]. The *STGD3* gene was named for elongation of very long chain fatty acids-4 (*ELOVL4)* and its protein product was proposed by Zhang et al. [2] to be a fatty acid elongase. Among the tissues tested, the gene was expressed in brain, retina, skin, and testis [3]. In the retina, *ELOVL4* was expressed at a high level in rod and cone photoreceptor cells in human, mouse, and monkey [2-5]. Karan et al. [6] created a transgenic mouse model of STGD3 disease that expressed human *ELOVL4* containing the 5 bp deletion. A retinal degeneration was observed in two lines that correlated with the level of expression of the transgene.

The ELO family of fatty acid elongases is involved in adding two-carbon units to existing fatty acids to produce fatty acids of chain lengths 16–38 [7]. These elongases are especially important in the production of long chain polyunsaturated fatty acids (LC-PUFA), whose short chain precursors must be obtained from the diet [8]. Docosahexaenoic acid (DHA), with 22 carbons and 6 double bonds (22:6n3), is a member of the n3 family of essential PUFA and the most abundant fatty acid found in retinal photoreceptor membranes [9]. Studies over four decades have demonstrated the critical role that DHA plays in the maintenance of normal structure and function in the retina [10-21]. Thus, when the *STGD3* gene locus was found to have high sequence homology with the well known ELO family of fatty acid elongases, the first prediction was that the mutation in *ELOVL4* would affect DHA metabolism [2]. Indeed, some evidence has been presented in humans [22,23] and mice [24] to support this point of view.

Mammals cannot synthesize DHA de novo and must therefore depend on a dietary source of preformed DHA or on elongation of shorter chain PUFA such as α-linolenic acid (ALA; 18:3n3) [8]. Significant changes in retinal DHA and other PUFA levels can be effected by dietary restriction of n3 PUFA, especially if started early in pregnancy and continued during the nursing period and thereafter [25,26]. However, this involves feeding two different diets to animals born at different times to different dams. Another approach to manipulating retinal PUFA levels was developed by Kang et al. [27], who created a transgenic mouse that expressed the *fat-1* gene, which encodes an n3 desaturase. Originally cloned from *Caenorhabditis elegans* [28], this enzyme efficiently converts n6 PUFA to n3 PUFA in all tissues of the body, making it possible to produce membrane lipids with vastly different n6:n3 ratios in mice from the same genetic background fed identical diets. We have recently used these mice to study the role of DHA in light damage susceptibility [29].

The purpose of the present study was to test the hypothesis that DHA slowed or prevented retinal degeneration in mice that express the mutant *hELOVL4* gene in rod and cone photoreceptor cells. We crossed a transgenic mouse line with a moderate rate of retinal degeneration (TG2 line [6]) with mice that expressed the *fat-1* gene and generated four predictable groups of experimental animals. All dams and offspring were fed the same diet, which had high levels of n6 and only traces of n3 PUFA (n6:n3 ratio=273 [29]). The results showed that high retinal levels of DHA did not protect the retinas of mice expressing the *ELOVL4* transgene from degeneration.

## Methods

### Animals

*Fat-1* transgenic male mice carrying a *fat-1* gene of *Caenorhabditis elegans* in a C57BL/6J background were kindly provided by Dr. Jing Kang (Department of Medicine, Massachusetts General Hospital and Harvard Medical School, Boston, MA). These mice were bred to heterozygous female mice generated by Karan et al. [6] in a C57BL/6J background that, before breeding, had been fed a modified AIN-76A Purified Rodent Diet containing 10% safflower oil (SFO; Dyets Inc., Bethlehem, PA). SFO is an edible seed oil consisting primarily of triglycerides of linoleic acid (LA; 18:2n6), and has very low levels of n3 PUFA and no DHA. The composition of this diet can be found in Tanito et al. [29]. Four different groups of mice, Fat1^+^/TG2^+^, Fat1^–^/TG2^+^, Fat1^+^/TG2^–^, and Fat1^–^/TG2^–^, were identified within the same group of litters by genotyping. The offspring were weaned at 3 weeks of age and maintained on the same SFO diet fed their parents. Animals were cared for and handled according to the Association for Research in Vision and Ophthalmology (ARVO) statement for the use of animals in vision and ophthalmic research. All protocols were approved by the Institutional Animal Care and Use Committee (IACUC) and comply with the guidelines of the University of Oklahoma Faculty of Medicine and the Dean McGee Eye Institute for use of animals in research.

### Genotyping

Fresh tail snips (2 mm) were digested overnight with DirectPCR tail lysis reagent (Viagen Biotech, Los Angeles, CA) containing 0.3 mg/ml of Proteinase K (Sigma, St Louis, MO) at 55 °C. The lysates were incubated at 95 °C for 5 min, and 2 μl of lysate was used per PCR reaction in Green Go Taq master mix (Promega, Fitchburg, WI). The *fat-1* transgene was detected using primers (5′-CTG CAC CAC GCC TTC ACC ACC C-3′ and 5′-ACA CAG CAG ATT CCA GAG ATT-3′), which amplify a 251 bp product. A separate PCR reaction was performed to detect the *TG2* transgene using primers (5′-TAA GTG GGT TGC AGG AGG AC-3′ and 5′-TTG GGG AAG GGG CAG TC-3′), which amplify a 215 bp product. PCR products were visualized on 1.25% agarose gels.

### Electroretinography

Animals kept in total darkness overnight were anesthetized with an intramuscular injection of 120 mg/kg bodyweight ketamine and 6 mg/kg bodyweight xylazine under dim red light. One drop of 10% phenylephrine was applied to each animal’s cornea to dilate the pupil and one drop of 0.5% proparacaine HCl was applied for local anesthesia. A reference electrode was positioned in the mouth and a ground electrode on the tail. Nine different flash intensities, ranging from 0.001 to 2000 cd.s/m^2^, were used. Electroretinogram (ERG) responses from both eyes were recorded with gold electrodes placed on the cornea (Espion E2 ERG System, Diagnosys, LLC, Lowell, MA). The a-wave and b-wave amplitudes from each eye were determined and averaged for comparison of retinal function.

### Outer nuclear layer thickness measurement

After ERG testing, anesthetized animals were euthanized by asphyxiation with carbon dioxide. Right eyes were enucleated, fixed with Perfix (20% isopropanol, 2% trichloroacetic acid, 4% paraformaldehyde, and 2% zinc chloride) and embedded in paraffin. Next, 5 µm thick sections were taken along the vertical meridian to allow comparison of all regions of the retina in the superior and inferior hemispheres. In each of the hemispheres, outer nuclear layer (ONL) thickness was measured in nine defined areas, starting at the optic nerve head and extending along the vertical meridian toward the superior and inferior ora serrata. Measurements were made at 225 µm intervals. Mean ONL thickness was then calculated for the entire retinal section.

### Lipid analysis

Fatty acid profiles were analyzed in whole retina, liver, and plasma from Fat1^+^/TG2^+^, Fat1^–^/TG2^+^, Fat1^+^/TG2^–^, and Fat1^–^/TG2^–^ mice of C57BL/6J strains. One single retina was taken for each analysis. Each plasma sample was obtained by centrifuging the heparinized blood at 2,000x g in EGTA-containing tubes to obtain approximately 100 µl plasma. For whole retina and plasma, total lipids were extracted following the method of Bligh and Dyer [30]. The tissues were extracted in chloroform:methanol:water (1:1:1) and the chloroform phase collected. The remaining aqueous phase was extracted once again with chloroform, keeping the chloroform:methanol:water ratios at 1:1:1. As before, the chloroform phase was collected and combined with the chloroform phase from the initial extraction. The combined chloroform phases were then extracted with chloroform:methanol:water (3:48:47) and the aqueous phase discarded. The remaining purified lipid extract was stored under nitrogen. For liver, total lipids were extracted following the method of Folch et al. [31]. The tissues (n=3–4 mice per group) were homogenized in 4 ml of chloroform:methanol (2:1). Proteins in the homogenate were pelleted by centrifugation at 1,000x g for 10 min and the lipid extract was removed. The pellet was washed twice with 1 ml of chloroform:methanol 1:1 and the washes were combined with the lipid extract. The lipid extract was then washed with 0.2 volumes of 1 mM DTPA(aq) followed by 0.2 volumes of chloroform:methanol:water (3:48:47), each time discarding the aqueous phase. The resulting purified lipid extract was dried under nitrogen and re-suspended in a known volume of toluene. Purified lipid extracts from plasma and liver were resolved into individual lipid classes using one-dimensional thin-layer chromatography. Briefly, an aliquot of each extract was spotted onto 10×20 cm Silica Gel 60 plates (EM Science, Gibbstown, NJ). Lipid classes were resolved hexane:etyhy ether: glacial acetic acid (70:30:2.3, by vol) acid mobile phase, and plates were stained with 0.05% (wt/vol) 2,7-dichlorofluorescein in 75% (vol/vol) methanol. Fatty acids of purified lipid extracts of whole retina and of scraped thin-layer chromatography spots from plasma and liver extracts were derivatized to form fatty acid methyl esters and analyzed using gas-liquid chromatography. The fatty acid compositions were determined by injecting 3 µl of each sample at 250 °C with a split ratio of 20:1 (10:1 for whole retina) onto a (30 m long x 0.32 mm I.D.) DB-225 capillary column (J&W Scientific, Folsom, CA) in an Agilent 6890N gas chromatograph with model 7683 autosampler (Agilent Technologies, Wilmington, DE). The column temperature was programmed to begin at 160 °C, ramped to 220 °C at 1.33 °C/min, and held at 220 °C for 18 min. Hydrogen carrier gas was allowed to flow at 1.6 ml/min, and the flame ionization detector temperature was set to 270 °C. The chromatographic peaks were integrated and processed with ChemStation® software (Agilent Technologies). FAMES were identified by comparison of their relative retention times with authentic standards (NU-CHEK PREP, Elysian, MN) and relative mole percentages were calculated.

### Rhodopsin assay

Mice were dark-adapted overnight and euthanized under dim red light by carbon dioxide asphyxiation. One eye was removed from each animal and homogenized in buffer containing 10 mM Tris (pH 7.4), 150 mM NaCl, 1mM EDTA, 2% (wt/vol) octylglucoside, and 50 μM hydroxylamine. Homogenates, maintained in dim red light, were centrifuged at 16,000x g, and the soluble fractions were scanned from 270 to 800 nm in an Ultrospec 3000 UV/Vis spectrophotometer (GE Healthcare, Piscataway, NJ). The samples were subsequently bleached by exposure to room light (approximately15 min) and scanned again. The difference in absorbance at 500 nm between unbleached and postbleached samples was used to determine rhodopsin content, using a molar extinction coefficient of 42,000 M^–1^ [32].

### Statistical analysis

Results are expressed as the mean±SD. Differences were assessed by multivariant ANOVA followed by Neuman-Keuls posthoc test. A p<0.05 was considered significant.

## Results

### Fatty acid composition of plasma, liver, and retina

**Figure 1:**
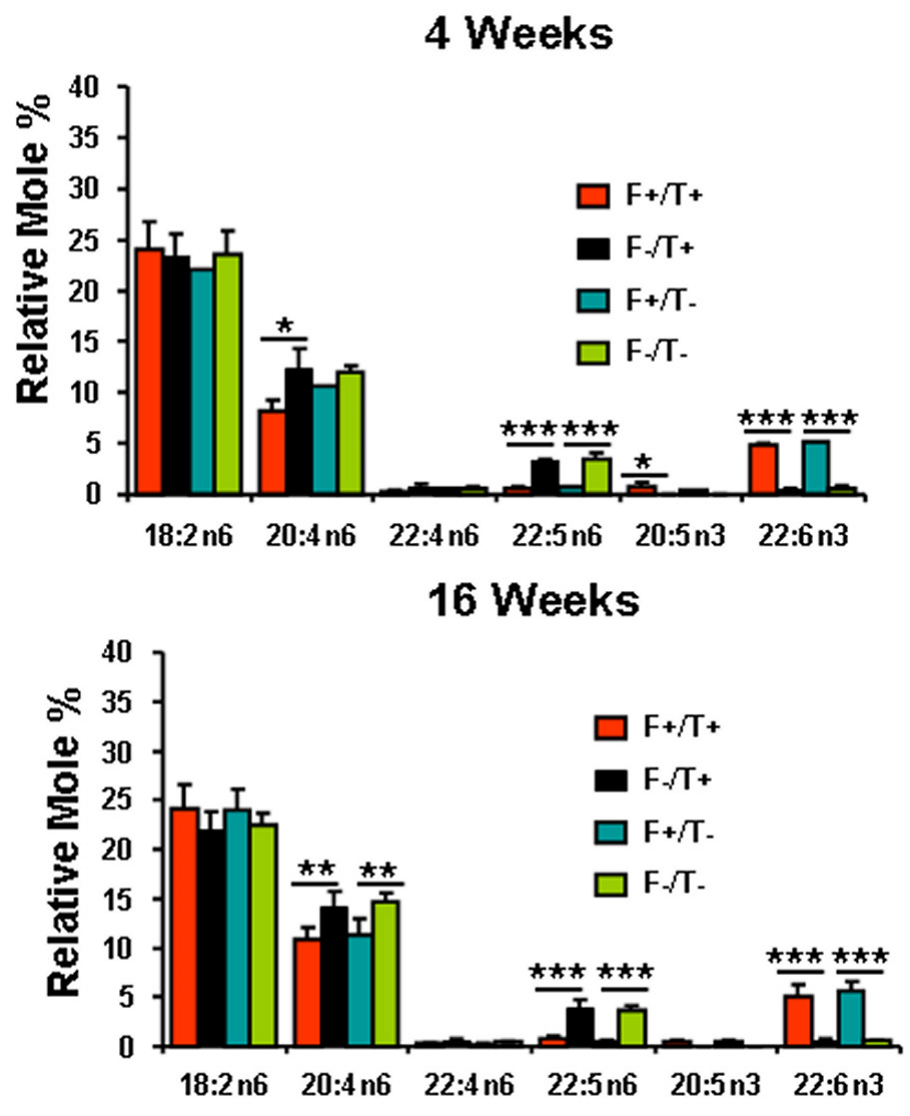
Plasma fatty acid composition. Relative mole percentage (±SD) of n3 and n6 polyunsaturated fatty acids are from total phospholipids of plasma of Fat1+/TG2+, Fat1–/TG2+, Fat1+/TG2–, and Fat1–/TG2– mice at age of 4 weeks (**A**; n=3–4) and 16 weeks (**B**; n=4–6). Single (*), double (**), and triple (***) asterisks indicate p<0.05, p<0.01, and p<0.001 for *fat-1* positive versus *fat-1* negative values. *p<0.05 *fat-1* positive versus *fat-1* negative. Double asterisk (**) indicates a p<0.01 *fat-1* positive versus *fat-1* negative. Triple asterisk (***) represents a p<0.001 *fat-1* positive versus *fat-1* negative.

**Figure 2:**
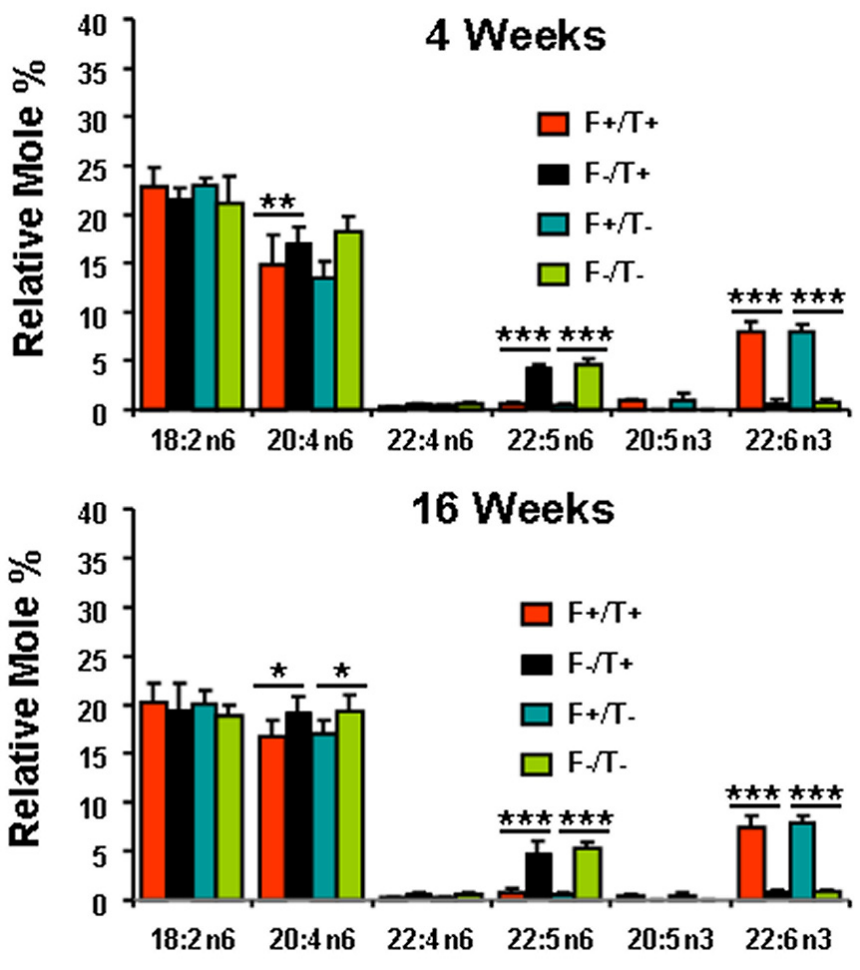
Liver fatty acid composition. Relative mole percentage (±SD) of n3 and n6 polyunsaturated fatty acids are from total phospholipids of plasma of Fat1+/TG2+, Fat1–/TG2+, Fat1+/TG2–, and Fat1–/TG2– mice at age of 4 weeks (**A**; n=4–5) and 16 weeks (**B**; n=4–7). Single (*), double (**), and triple (***) asterisks indicate p<0.05, p<0.01, and p<0.001 for *fat-1* positive versus *fat-1* negative values Single asterisk (*) indicates a p<0.05 *fat-1* positive versus *fat-1* negative. Double asterisk (**) represents a p<0.01 *fat-1* positive versus *fat-1* negative. Triple asterisk (***) indicates a p<0.001 *fat-1* positive versus *fat-1* negative.

**Figure 3:**
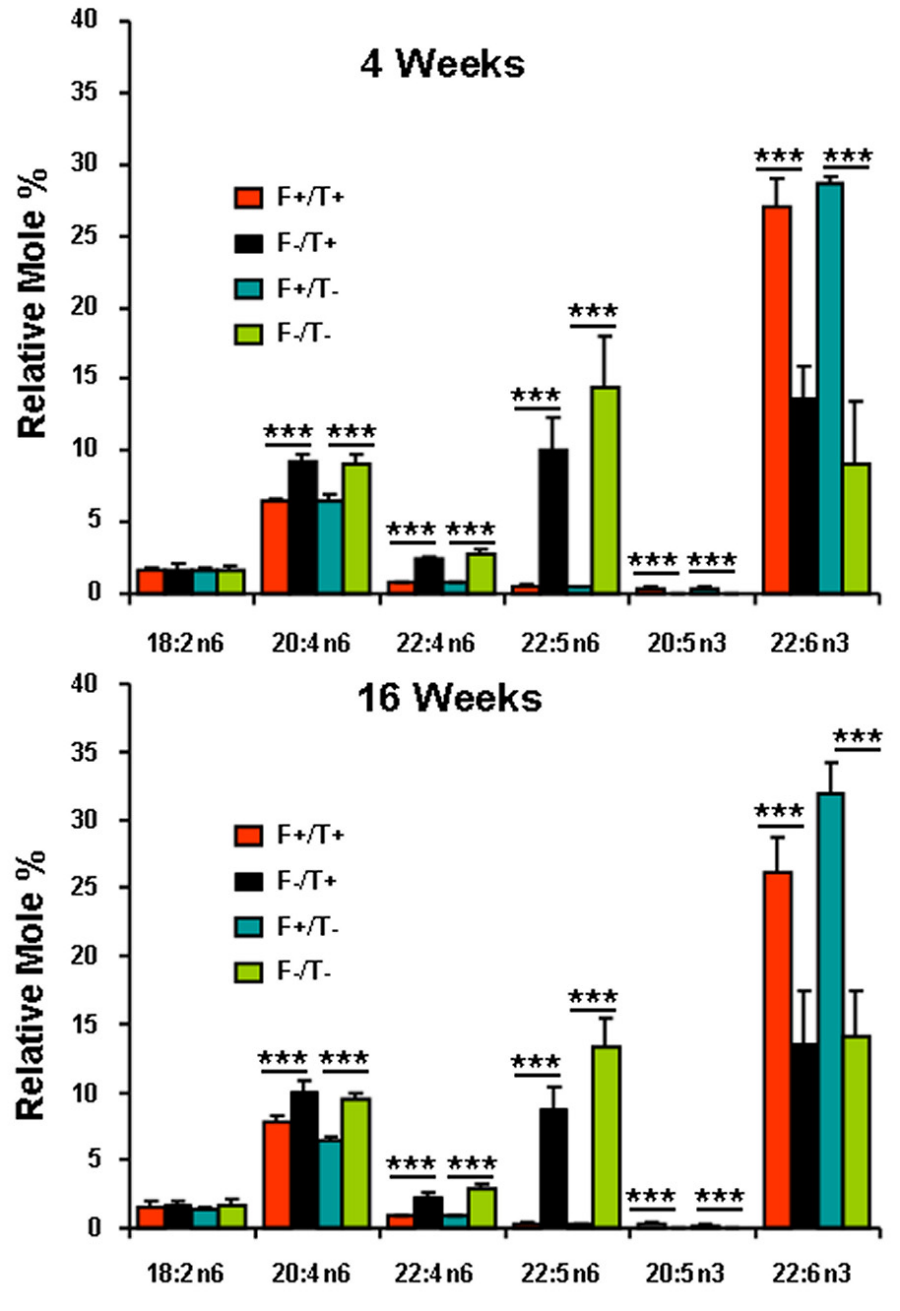
Retina Fatty Acid Composition. Relative mole percentage (±SD) of n3 and n6 polyunsaturated fatty acids are from total phospholipids of plasma of Fat1+/TG2+, Fat1–/TG2+, Fat1+/TG2–, and Fat1–/TG2– mice at age of 4 weeks (**A**; n=3–5) and 16 weeks (**B**; n=5–6). Single (*), double (**), and triple (***) asterisks indicate p<0.05, p<0.01, and p<0.001 for *fat-1* positive versus *fat-1* negative values.

Mice expressing the *fat-1* gene can efficiently convert n6 to n3 PUFA in all tissues that express the gene. Since none of the dams expressed *fat-1* and all were fed the SFO diet before conception, only the pups expressing *fat-1* were able to synthesize n3 PUFA. This allowed the development of a model in which membrane PUFA content could be manipulated in littermates fed the same diet thus reducing variability due to differences in diet and genetic background. Figure 1 and Figure 2 show the fatty acid compositions of the total phospholipids from plasma and liver, respectively, while Figure 3 shows the fatty acid composition of the total lipid extract from retina. In general, the mice expressing *fat-1* had higher percentages of 22:6n-3 (p<0.001) and lower percentages of 20:4n-6 (p<0.05 to approximately 0.01), 22:4n-6 (p<0.001), and 22:5n-6 (p<0.001); 20:5n-3 could not be detected in the *fat-1* negative mice. These differences were already established by 4 weeks of age and did not change appreciably over the ensuing 12 weeks. The expression of the *TG2* transgene did not affect the fatty acid composition of any of the three tissues that were examined.

The levels of retinal DHA in the *fat-1* negative mice were roughly half of that in *fat-1* expressing mice (Figure 3). As reported previously by many laboratories, reduction in DHA in the retina was accompanied by a near-equivalent increase in 22:5n6 [11,18,19,33-35]. Even though the dams were fed a SFO diet before conception, the retinas of the *fat-1* negative mice still contained relatively high levels of n3 PUFA. This is not surprising, since neural tissues accumulate n3 PUFA from their mothers in utero and during the 3-week nursing period before weaning. Maintenance of these levels of n3 PUFA at 16 weeks of age is consistent with earlier studies showing that the retina has an efficient mechanism of conserving n3 PUFA by recycling from the retinal pigment epithelium [25].

### Effect of DHA levels on retinal structure

**Figure 4:**
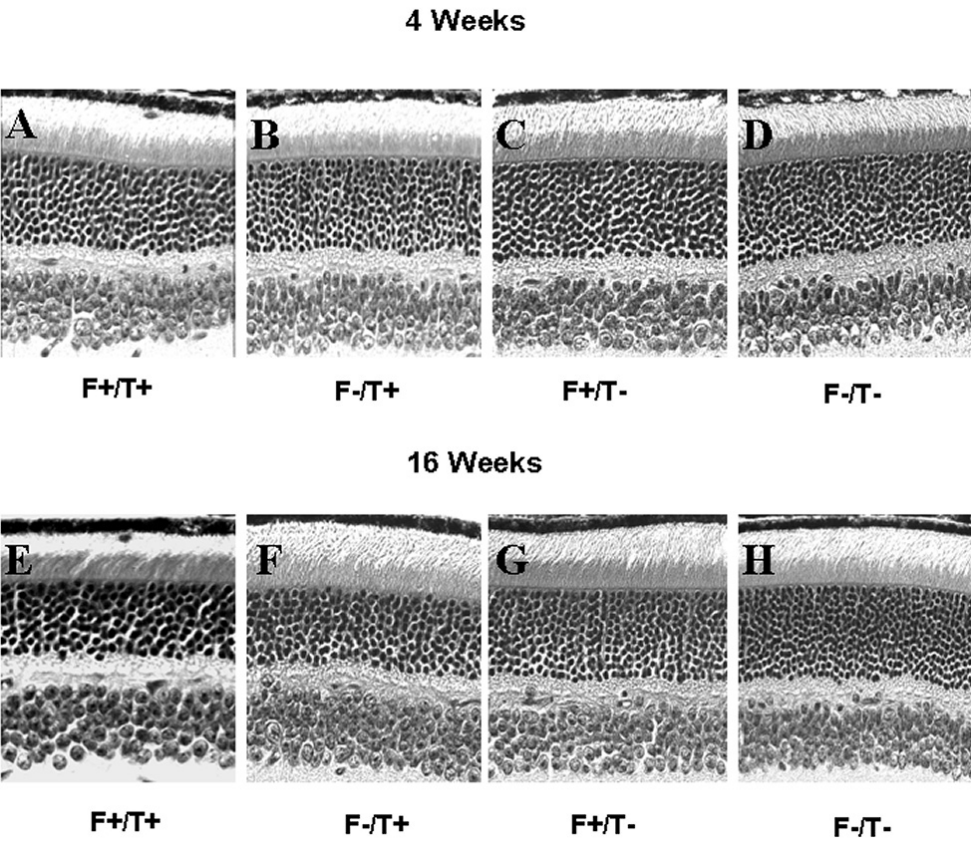
Retinal structure in transgenic mice. Representative photomicrographs are from the central superior retina of Fat1+/TG2+, Fat1–/TG2+, Fat1+/TG2–, and Fat1–/TG2– mice at 4 weeks (**A-D**) and 16 weeks of age (**E-H**).

Retinal structure was examined along the vertical meridian from inferior to superior ora serrata; representative sections from central superior retina are shown in Figure 4. At 4 weeks of age, there was a slight loss of rod photoreceptors in *TG2* positive (T^+^) mice that also expressed *fat-1* (F^+^; Figure 4A). By 16 weeks of age, there was a significant thinning of the ONL of both T^+^ groups compared to the T^–^ groups. The presence of higher levels of DHA in the T^+^/F^+^ group (Figure 4A,E) had no effect on rod photoreceptor survival.

**Figure 5:**
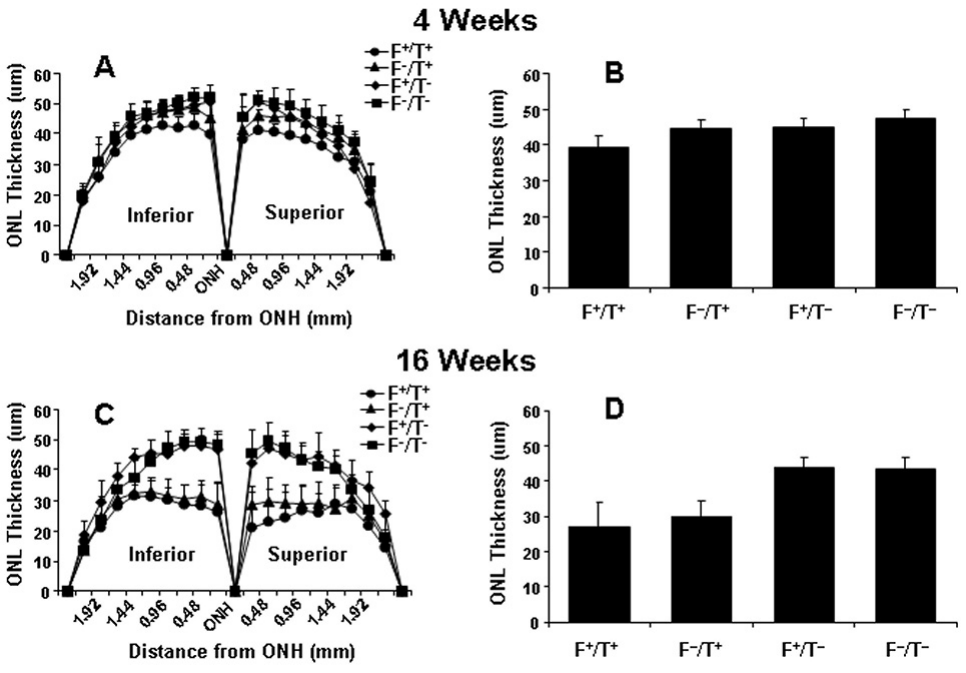
Quantification of morphologic changes in transgenic mice. Measurements of ONL thickness (±SD) were made along the vertical meridian of the eye in Fat1+/TG2+, Fat1–/TG2+, Fat1+/TG2–, and Fat1–/TG2– mice maintained in 20 lx cyclic light (n≥8). **A**, **C:** Averages were made of ≥8 retinas of transgenic mice at 4 and 16 weeks of age. **B**, **D:** The histograms are the average ONL thickness at both age groups.

Quantification of age-related loss of rod photoreceptors at each point measured along the vertical meridian is presented in the “spider” graphs in Figure 5A,C. Photoreceptor nuclei were lost in both inferior and superior regions of the mice expressing the *ELOVL4* transgene compared to controls, especially at 16 weeks of age. Mean ONL thicknesses taken from 14 sites along the vertical meridian (7 superior and 7 inferior of the optic nerve head) were calculated for both age groups. There were no significant differences in ONL thickness between F^+^/T^+^ versus F^–^/T^+^ and F^+^/T^–^ versus F^–^/T^–^ mice at 4 and 16 weeks of age. However, compared to 4-week-old T^+^ mice (Figure 5B), ONL thickness was reduced 31% in F^+^ mice and 32% in F^–^ mice at 16 weeks of age (Figure 5D).

### Effect of DHA levels on retinal function

**Figure 6:**
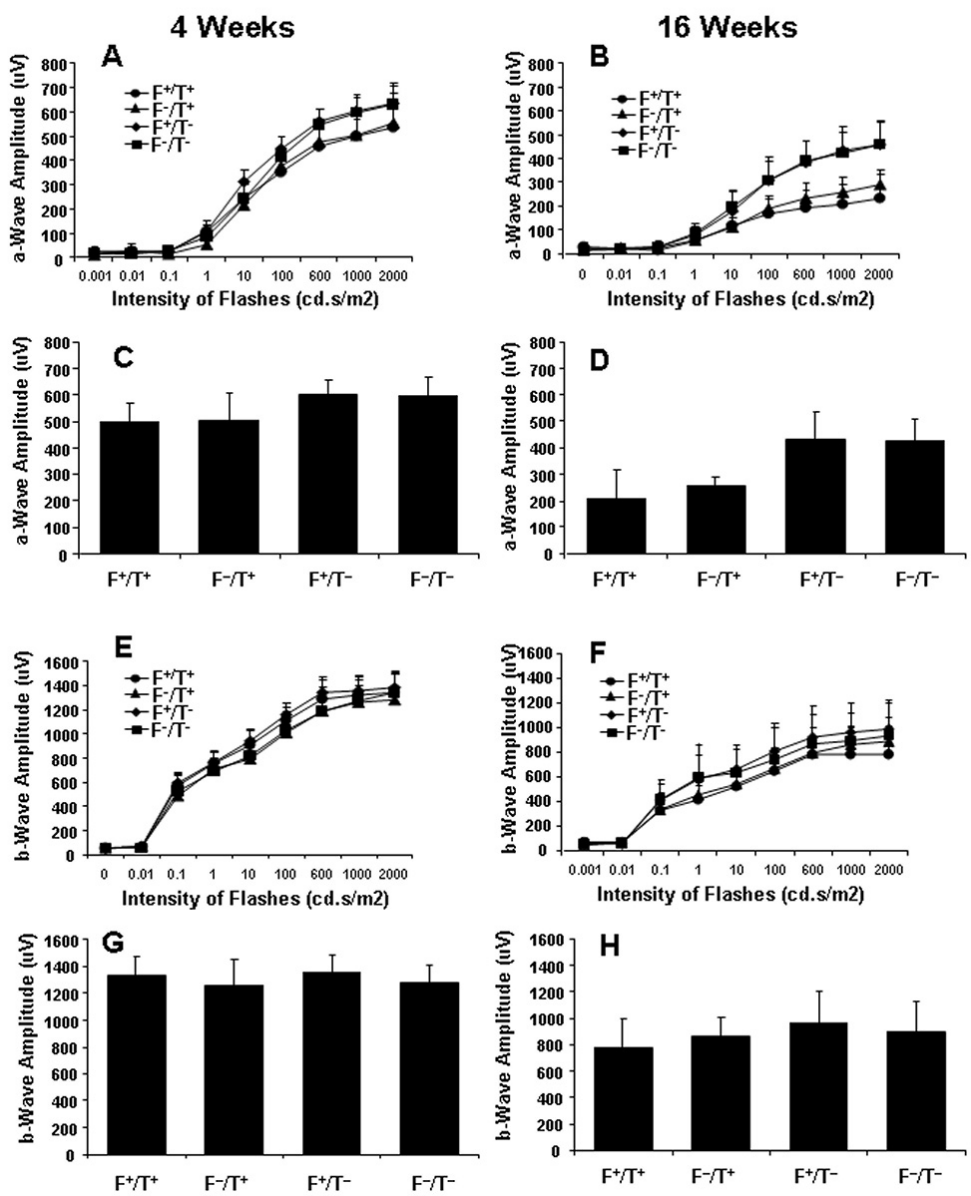
Evaluation of functional changes by electroretinography. Measurements of ERG a- and b-wave amplitudes (±SD) were made in Fat1+/TG2+, Fat1–/TG2+, Fat1+/TG2–, and Fat1–/TG2– mice maintained in 20 lx cyclic light (n≥10). The a-wave (**A**, **B**) and b-wave (**E**, **F**) amplitudes are from nine flash intensities at 4 weeks and 16 weeks. **C, D** are the average a-wave amplitudes of transgenic mice at 4 weeks and 16 weeks of age. **E**, **H** are b-wave amplitudes.

ERG was used to detect retina function for both age groups. Nine different intensity flashes, ranging from 0.001 to 2,000 cd.s/m^2^, were used (Figure 6A,B,E,F) and ERG responses from both eyes were recorded and averaged. The amplitudes of mice at saturating flash intensity, 1,000 cd.s/m^2^, are shown in Figure 6C,D,G,H. As expected, ERG a-wave responses were reduced at 16 weeks of age in the mice expressing the *ELOVL4* transgene, reflecting the significant degeneration observed by histology and morphometry. However, we observed no effect of DHA levels on the response amplitudes of the ERG of T^+^ or T^–^ mice. The amplitudes of a-waves in the T^+^ mice were already slightly lower than those in T^–^ mice at 4 weeks of age (Figure 6A,C), although there were no differences in the b-waves (Figure 6E,G). There was a significant age-related reduction in both a-wave and b-wave amplitudes of all four groups (Figure 6). Interestingly, the a-wave amplitudes, but not the b-wave amplitudes, of 16-week-old T^+^ mice were lower than those of T^–^ mice.

### Effect of DHA on rhodopsin levels

**Figure 7:**
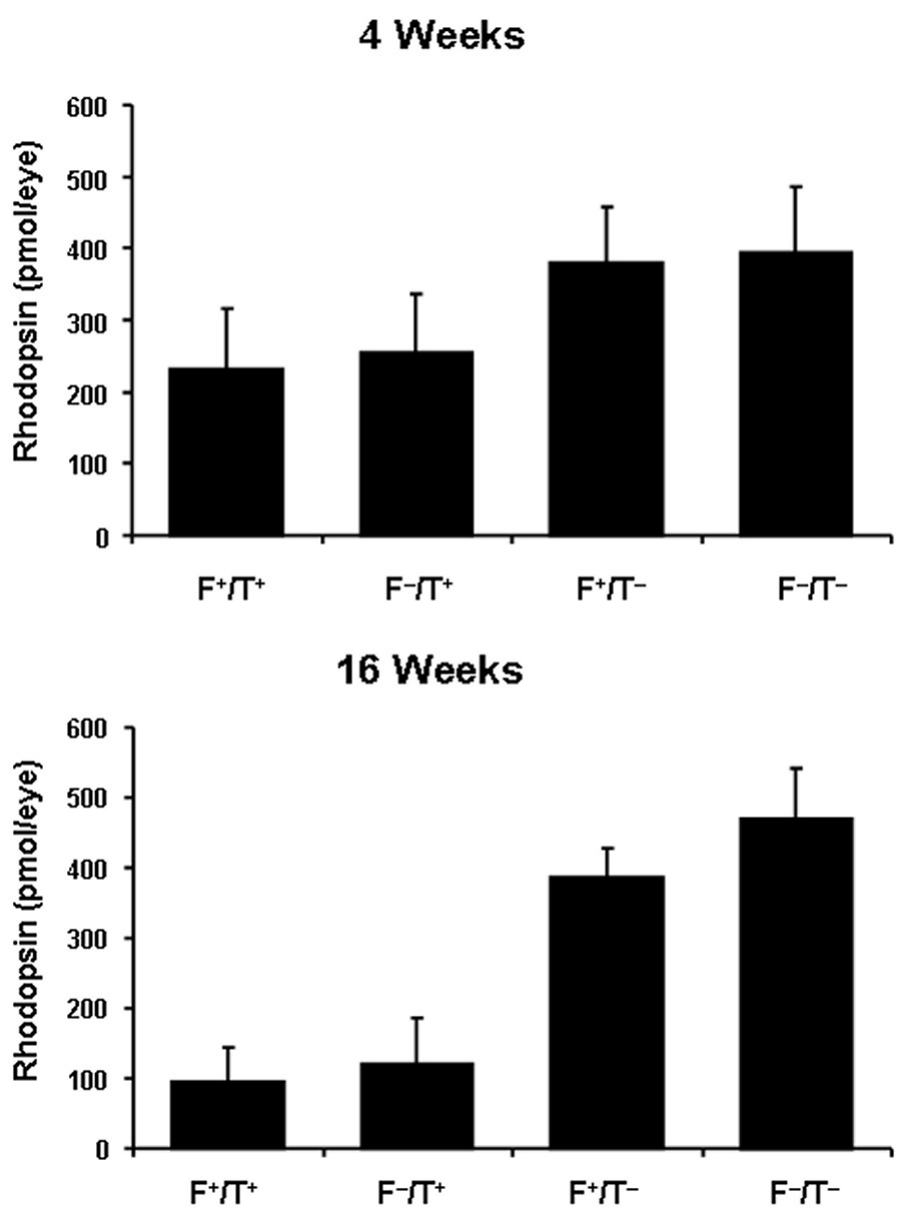
Rhodopsin content in transgenic mice. Rhodopsin content was determined in eyes (±SD, n≥4) from Fat1+/TG2+, Fat1–/TG2+, Fat1+/TG2–, and Fat1–/TG2– mice at age of 4 weeks (**A**) and 16 weeks (**B**).

Figure 7 shows the levels of bleachable rhodopsin in the four experimental groups of mice. There was already some reduction in rhodopsin levels at 4 weeks of age in the T^+^ mice compared to T^–^ mice. This reduction was even more dramatic at 16 weeks of age, corresponding to the loss of photoreceptors demonstrated by our ONL thickness measurements (Figure 5). However, as was the case in our morphometric and functional analyses, DHA levels had no effect on the rhodopsin content in T^+^ or T^–^ mouse retinas.

## Discussion

The *fat-1* transgene provides the opportunity to significantly alter the membrane content of n3 and n6 PUFA in animals derived from the same dam and fed the same diet [29]. In the current study, we produced mice with dramatically different retinal DHA levels that also expressed the transgene encoding one of the *ELOVL4* mutations found in patients with STGD3 [6]. Mice expressing the *fat-1* transgene, fed an n3-deficient SFO diet, produced large amounts of n3 PUFA, resulting in significantly higher levels of DHA in retina, liver, and plasma as was found in our earlier light damage study [29]. As expected, retinas showed the highest level of DHA. Mice without *fat-1* expression fed the SFO diet had fatty acid profiles similar to those reported in other studies of n3 dietary deficiency [11,18,19,33-35], namely a decrease in DHA that was offset by an increase in 22:5n6. It is interesting that the retinas of *fat-1* negative mice (wild type) had relatively high levels of DHA (although still half those of the *fat-1* positive mice), even though their dams had been fed the SFO diet well before conception. This illustrates the striking ability of the retina and the retinal pigment epithelium to sequester DHA from the blood and to conserve it in the retina by a highly efficient recycling system between the two tissues [25,36].

The gene mutation responsible for autosomal dominant Stargardt macular dystrophy, first described in 2001 [2], was found to have significant sequence homology to a family of fatty acid elongases [2], all of which catalyze the condensing reaction responsible for 2-carbon elongation of fatty acids. These reactions occur on the endoplasmic reticulum and each ELOVL enzyme (including ELOVL4) has a C-terminal endoplasmic reticulum retention motif [7]. The265 mutated *ELOVL4* gene product is truncated and no longer contains the endoplasmic reticulum signal [37-39], so it is possible that the effect of the mutation is interference with fatty acid elongation in the retina and other tissues in which the gene is expressed. It was suggested that the metabolic defect in STGD3 might be in the elongation of shorter chain n3 PUFA to DHA [2], since it is the major fatty acid in the retina and rod outer segments [9], and numerous studies in animals [10,11,14-19,21,34,40] and humans [12,13,20] have shown that DHA is essential for optimal development and function of the retina. We now know that ELOVL4 is not involved in DHA synthesis, as we recently showed that it catalyzes the elongation of C26 saturated and polyunsaturated fatty acids to C28-C38 fatty acids [41]. Nevertheless, it remained to be determined if retinal DHA also played a role in the retinal degenerative process that occurs in STGD3, since two studies in patients had suggested a potentially beneficial role [22,23]. The current study is also important since the AREDSII clinical trial has DHA and eicosapentaenoic acid (20:5n3) supplementation as one of its variables.

There was a significant reduction in the amplitudes of the a-waves and b-waves of the ERG as a function of age (Figure 6), with the greatest relative reduction occurring in the a-wave. A similar result was reported by Li et al. [42] in C57BL/6 mice at ages 2, 6, and 12 months. Although not discussed, there appeared to be a relatively greater loss of a-wave amplitude in their study as well. They could not explain the loss, but suggested that it could be due to decreased resistance as a function of age. This explanation seems reasonable, since the ERG is a measure of electrical activity of the retina, and significant growth occurs between 4 and 16 weeks—the age of the mice used in our study. Lee and Flannery [43] also noted an age-related reduction in ERG amplitudes in heterozygous rhodopsin knockout mice, with the greatest relative change occurring in the a-wave. In our study, we fed a special diet devoid of n3 PUFA. However, our observations of ERG changes is not likely related to diet, since the observations of Li et al. [42] and Lee and Flannery [43] were made on animals fed laboratory chow diets.

The results of our study show that high DHA levels in the retinas of mice overexpressing the mutated *ELOVL4* transgene do not slow or prevent the retinal degeneration. Although the retinal levels of DHA in the *fat-1* positive mice were twice those of the wild-type animals fed an n3-deficient diet, there was no effect of DHA on the structure or function of the retina. We therefore conclude that in the animal model we used, namely a transgenic mouse overexpressing mutant human *ELOVL4* along with two normal mouse copies, DHA does not rescue structure or function of rod photoreceptors.
